# THERAPEUTIC CANNABIS USE: UNIQUE CONTEXTS FOR OLDER ADULTS

**DOI:** 10.1093/geroni/igad104.0563

**Published:** 2023-12-21

**Authors:** Pinchas Cohen

## Abstract

Nationwide cannabis use prompts new questions about its therapeutic use. Self-administered cannabis among older adults demands careful study on their treatment outcomes and consumption patterns, in light of a more accessible, legalized cannabis market. Changing sentiments toward the illicit drug urges new research into its viability as a treatment for conditions in older adults. Ailshire uses the 2018-2021 Behavioral Risk Factor Surveillance System (BRFSS) surveys to compare cannabis use by age group. The data, which is representative of 39 U.S. states, indicate that medical marijuana use increases with age. Furthermore, Kaskie’s Cannabis and Older Persons Study (COPS) uncovers the influence of age on cannabis use and motives for consumption. The study is grounded in three core objectives: distinguishing senior consumers from other age groups, distinguishing therapeutic and recreational use, and distinguishing life-time and naive cannabis consumers. With the support of the National Cancer Institute, Fahey conducts a cross-sectional study on consumption patterns of cancer patients over the age of 65, analyzing patient reported outcomes of symptom management through cannabis. Finally, Bluthenthal and Cohen perform quantitative, health surveys among 429 community-recruited people who inject drugs (PWID) to understand the potential benefit of a legal cannabis as a substitution for criminalized substances and alcohol. The papers in this symposium provide updated insights into widespread cannabis use among older adults. These findings may help forward conversations about the political and clinical changes that must be made in order to protect the therapeutic needs of older adults amidst a rapidly expanding cannabis industry.

